# The chromosome 9 ALS and FTD locus is probably derived from a single founder

**DOI:** 10.1016/j.neurobiolaging.2011.08.005

**Published:** 2012-01

**Authors:** Kin Mok, Bryan J. Traynor, Jennifer Schymick, Pentti J. Tienari, Hannu Laaksovirta, Terhi Peuralinna, Liisa Myllykangas, Adriano Chiò, Aleksey Shatunov, Bradley F. Boeve, Adam L. Boxer, Mariely DeJesus-Hernandez, Ian R. Mackenzie, Adrian Waite, Nigel Williams, Huw R. Morris, Javier Simón-Sánchez, John C. van Swieten, Peter Heutink, Gabriella Restagno, Gabriele Mora, Karen E. Morrison, Pamela J. Shaw, Pamela Sara Rollinson, Ammar Al-Chalabi, Rosa Rademakers, Stuart Pickering-Brown, Richard W. Orrell, Michael A. Nalls, John Hardy

**Affiliations:** a Reta Lila Weston Research Laboratories, Departments of Molecular Neuroscience and of Clinical Neuroscience, UCL Institute of Neurology, Queen Square, London, UK; b Molecular Genetics Section and Neuromuscular Diseases Research Group, Laboratory of Neurogenetics, National Institute on Aging, NIH, Bethesda, MD, USA; c Helsinki University Central Hospital, Department of Neurology, Molecular Neurology Research Program, Biomedicum, University of Helsinki, Helsinki, Finland; d Department of Pathology, Haartman Institute, University of Helsinki and HUSLAB, and Folkhalsan Institute of Genetics, Helsinki, Finland; e Department of Neuroscience, University of Turin, and Azienda Ospedaliera Universitaria San Giovanni Battista, Turin, Italy; f Medical Research Council Centre for Neurodegeneration Research, King's College London, Institute of Psychiatry, London, UK; g Department of Neurology, Mayo Clinic, Rochester, MN, USA; h Memory and Aging Center, Department of Neurology, University of California, San Francisco, San Francisco, CA, USA; i Department of Neuroscience, Mayo Clinic, Jacksonville, FL, USA; j Department of Pathology and Laboratory Medicine, University of British Columbia, Vancouver, BC, Canada; k Medical Research Council Centre for Neuropsychiatric Genetics and Genomics, Cardiff University School of Medicine, Cardiff, UK; l Department of Clinical Genetics, Section of Medical Genomics, VU University Medical Centre, Amsterdam, The Netherlands; m Department of Neurology, Erasmus Medical Center, Rotterdam, The Netherlands; n Molecular Genetics Laboratory, Azienda Ospedaliera OIRM-Sant'Anna, Turin, Italy; o Fondazione Salvatore Mangeri, IRCCS Scientific Institute of Milan, Milan, Italy; p School of Clinical and Experimental Medicine, University of Birmingham, and Queen Elizabeth Hospital, University Hospitals Birmingham NHS Foundation Trust, Birmingham, UK; q The Sheffield Institute for Translational Neuroscience (SITraN, Department of Neuroscience, University of Sheffield, Sheffield, UK; r Neurodegeneration and Mental Health Research Group, Faculty of Human and Medical Sciences, University of Manchester, Manchester, UK; s Molecular Genetics Section, Laboratory of Neurogenetics, National Institute on Aging, National Institutes of Health, Bethesda, MD, USA

**Keywords:** Genetics, Amyotrophic lateral sclerosis, Frontotemporal dementia, Finland

## Abstract

We and others have recently reported an association between amyotrophic lateral sclerosis (ALS) and single nucleotide polymorphisms on chromosome 9p21 in several populations. Here we show that the associated haplotype is the same in all populations and that several families previously shown to have genetic linkage to this region also share this haplotype. The most parsimonious explanation of these data are that there is a single founder for this form of disease.

## 1 Introduction

Amyotrophic lateral sclerosis (ALS) is a fatal neurodegenerative disease affecting motor neurons characterized by rapidly progressive weakness and ultimately death from respiratory failure typically within 3 years of symptom onset. Understanding the genetic etiology of the disease has been a focus for the ALS research community, as each new gene provides fundamental insights into the pathogenesis of motor neuron degeneration, as well as accelerating disease modeling and the design and testing of targeted therapeutics.

Using a genome-wide association study (GWAs) approach, we recently reported that a locus on chromosome 9p21 accounted for > 40% of familial ALS and nearly 1-fourth of all ALS cases in a sample of 405 Finnish patients (Laaksovirta et al., 2010). This association signal had previously been reported by van Es et al. (2009) as showing association with ALS and a meta-analysis amongst many studies showed that this was indeed the major signal for this disease (Shatunov et al., 2010). Similarly, a recent GWAs for frontotemporal dementia (FTD) with TDP-43 pathology had also identified this locus (Van Deerlin et al., 2010).

Linkage analysis of kindreds affected with multiple cases of ALS, FTD, and FTD-ALS with type 2 TDP-43 pathology had suggested there was an important locus for the disease on chromosome 9p (Boxer et al., 2011; Morita et al., 2006; Pearson et al., 2011; Vance et al., 2006) but it had not been clear whether the linkage and association signals related to a single locus or whether the different studies were reporting the same alleles at that locus.

The analysis in the Finnish population had narrowed the association signal to a 232 kb block of linkage disequilibrium, and allowed the identification of a haplotype that increased risk of disease by over 20-fold. Despite considerable efforts in our laboratories and others the underlying causative variant and deleterious mutation has not yet been identified.

Here, we examine the prevalence of the Finnish risk haplotype in other European populations to determine its geographical distribution and to analyze the possibility that it represents a founder mutation. We then tested this haplotype in ALS and FTD families with evidence of linkage to this region to determine if the same haplotype is responsible for both ALS and FTD. In the 4 families for which we had access to primary genetic data, the haplotype was consistent with the Finnish one.

## 2 Methods

We analyzed GWAs data obtained for ALS patients and neurologically normal controls in 5 populations in which we have access to the raw genotype data. These are the Finnish dataset (Laaksovirta et al., 2010), the Irish dataset (Cronin et al., 2008), the UK dataset (Shatunov et al., 2010), the US dataset (Schymick et al., 2007), and the Italian dataset (Chiò et al., 2009). All cohorts had been genotyped using Illumina (San Diego, CA, USA) single nucleotide polymorphism (SNP) arrays. Standard quality control procedures were applied to each dataset prior to combining summary statistics for meta-analysis. In brief, samples were excluded if they had call rates less than 95%, phenotype-genotype gender discordance, demonstrated cryptic relatedness (defined as pi_hat greater than 12.5%, effectively removing all first or second degree relatives), or outliers from the populations with European ancestry (defined as > 3 standard deviations away from the combined European Caucasians [HapMap 3 release 3, 2010 (International HapMap 3 Consortium, 2010)] population mean in components vectors 1 and 2, using PLINK Multidimensional scaling plot). SNPs were excluded if they had a minor allele frequency (MAF) < 0.01, Hardy-Weinberg equilibrium *p*-value < 10^−6^ in controls, missing by haplotype *p* value < 10^−4^, or evidence of nonrandom missingness in cases versus controls (*p* value < 10^−4^). Meta-analyses were performed with METAL (Willer et al., 2010) for fixed-effect and PLINK (Purcell et al., 2007) for random-effects model. Haplotype analysis was performed using Haploview 4.2 to evaluate the possibility of population-based differences (Barrett et al., 2005). Additional statistical analyses were performed using R (version 2.11.1, R Development Core Team, 2010). Subsequently, we tested families where phased genotype data generated on various SNP chips was available to establish the relationship between the 9p21 susceptibility region and the Mendelian linkage regions.

## 3 Results

We performed a meta-analysis of 5 ALS genome-wide association studies involving a total of 2017 cases and 3639 controls drawn from the 5 datasets. As expected, meta-analysis confirmed the presence of previously identified locus on chromosome 9p21 (most significantly with imputed SNP = rs2477521, *p* value = 4.51 × 10^−11^ based on fixed-effect model with heterogeneity *p* value of 1.5 × 10^−4^, and an overall *p* value = 0.00876 based on more conservative random-effects model). Heterogeneity estimates suggested significant variation in the effect size from different populations, with the Italian population being a frequent outlier. Secondary analysis without the Italian cohort yielded a markedly more robust *p* value for the same SNP (*p* value for rs2477521 under the fixed effect model = 1.24 × 10^−13^; rs10967973 with *p* value under the random effects model = 1.55 × 10^−10^). This suggests that the effect at this SNP differs markedly when comparing between populations of Northern and Southern European ancestry.

The original risk haplotype identified within the Finnish ALS population consisted of 42 SNPs stretching over a 232 kb region of chromosome 9p21 (Laaksovirta et al., 2010). This block of linkage disequilibrium was shorter in the European Caucasians HapMap data (24 SNPs over a 140 kb region), as would be expected in an outbred European population compared with the genetically homogeneous Finnish population (Shifman and Darvasi, 2001). Of these 24 SNPs, only 21 had been genotyped in all 5 populations. Furthermore, the most centromeric SNP of these 21 (rs1444533) did not show convincing association with disease in either the UK or Irish population. This SNP was therefore dropped from subsequent analysis, leaving a 20 SNP risk haplotype common to all Northern European ancestry groups in this meta-analysis. Thus, we restricted subsequent analyses to the region chr9: 27467874-27579657 (NCBI36/hg18) between SNP rs1444533 and rs696826.

This 20 SNP haplotype was associated with disease in Finland, was less significantly associated in the UK and US populations (Table 1); this haplotype had only a trend toward association in the Irish population (*p* = 0.17) and showed no evidence of association at all in the Italian population. The 20 SNP haplotype is consistent with the association recently reported for both FTD (Van Deerlin et al., 2010; Rollinson et al., 2011; see Table 2) and for ALS in a Dutch study although the incompleteness of the published data in these studies precludes a formal comparison.

Analysis of SNP chip data in 4 families with FTD or FTD-ALS (Boxer et al., 2011; Pearson et al., 2011; Seelaar et al., 2011; Traynor and Hardy, unpublished) in which linkage data was generated using SNP chips (Table 2) revealed that a similar disease haplotype was found in all patients with the exception of the most distal SNP (rs2477518) in the family reported by Seelaar et al. (2011). These data suggest that the same conserved chromosome 9p21 20 SNP risk haplotype underlies both ALS and FTD in multiple populations and that a proportion at least of the families showing genetic linkage to the region also share this rather short haplotypic region.

## 4 Discussion

These results are consistent with a single haplotype being associated with ALS, FTD, and FTD-ALS in most of the populations studied with the strength of the association being strongest in populations from Northern Europe that exhibit some estimated degree of Scandinavian ancestry and progressively weaker as one moves south and the contribution of this ancestral background is reduced. This interpretation is also consistent with the data from van Es et al. (2009) who first identified this association and showed a stronger association in a Swedish population than in the others included in their analysis (note that this analysis partially overlaps with our analysis reported here). This haplotype has the structure shown in Table 2 and extends over 140 kb and 3 genes *MOBKL2B*, *C9orf72*, and *IFNK*. Although this is the simplest explanation it is worth considering what other explanations would be consistent with the data. One such explanation is that the haplotype carries a premutation (such as an expanded repeat) which is predisposed to give rise independently to pathogenic alleles of differing penetrances.

The observations described above have several implications. First, if only a single founding haplotype bears the mutation this suggests that all, or at least the majority of individuals, with the disease possess the same pathogenic variant. Second, the lack of pathogenic coding mutations in the known genes within this locus suggests that the mutation(s) is of an unusual type involving something other than a simple missense or nonsense change. Possibilities would include inversions similar to the MAPT H2 haplotype, or the inclusion of cryptic exons or the exclusion of exons caused by variants distant from splice sites. Third, it seems that the same associated haplotype is found in both FTD and ALS. In this latter regard, it is interesting that, whereas a founder mutation of the MAPT gene largely explains the Manchester focus of FTD (Pickering-Brown et al., 2002) the well documented Lund focus of FTD in Sweden remains unexplained. Fourth, our data are consistent with the same haplotype being responsible for the disease in families showing linkage to this region suggesting they harbor the same pathogenic mutation: certainly this is the case in those families to which we have access. By explicitly publishing this haplotype, our data will enable those who have access to other families to assess whether this same haplotype is present in their families. It remains unclear as to why the apparent penetrance of the haplotype appears to be so variable. It could be that this reflects ascertainment bias, or that there have been subsequent additional variants accrued onto this ancient haplotype, or it could be that there is another epistatic locus elsewhere in the genome which influences penetrance as Gijselinck and colleagues have suggested (2010).

Clearly, the identification of this locus remains a major goal of ALS and FTD research. Our data suggest that this will be a difficult task and will require complete sequencing of the locus and of all the transcripts emanating from it.

## Disclosure statement

The authors disclose no conflicts.

Appropriate Ethical Committee approvals were in place for this work.

## Figures and Tables

**Table 1:** Twenty SNP haplotype frequencies in cases and controls

Population	Number (case:control)	Frequency		*p*
		Case	Control	
Finland	405:497	0.23	0.098	3.169E-14
Ireland	221:211	0.147	0.116	0.1716
Italy	500:247	0.129	0.149	0.3183⁎
UK	620:1890	0.182	0.135	9.17E-05
USA	271:794	0.145	0.111	0.0443

Haplotype frequencies in cases and controls and the *p*-value (χ^2^) for the nominal significance of the difference between them. The 20 SNPs are: rs1822723, rs4879515, rs868856, rs7046653, rs1977661, rs903603, rs10812610, rs2814707, rs3849942, rs12349820, rs10122902, rs10757665, rs1565948, rs774359, rs2282241, rs1948522, rs1982915, rs2453556, rs702231, and rs696826. Key: SNP, single nucleotide polymorphism.

⁎ In contrast to the other populations, the Italian cases have a marginally decreased frequency of the risk haplotype.

**Table 2:** The 24-SNP Finnish haplotype compared with 20 SNP haplotype and data from other populations, families, and publications

SNP	Position on ch9	This study					Consensus 20 SNP haplotype	Previous association studies			Data from families			
		Finnish	Irish	US	UK	Italian		Van Es et al. (2009)	Van Deerlin et al. (2010)	Rollinson et al. (2011)	Boxer et al. (2011)	Seelaar et al. (2011)	Pearson et al. (2011)	US number 3 (Traynor and Hardy, unpublished)
rs1444533	27467874	A	A	A	A	A	A^a^	—	—	—	A	A	T	A
**rs1822723**	27468052	C	C	C	C	C	C	—	—	—	C	C	—	—
**rs4879515**	27472235	T	T	T	T	T	T	—	—	—	T	T	T	T
rs895023	27473959	T	—	T	T	T	—	—	—	T	T	T	T	T
**rs868856**	27479251	T	T	T	T	T	T	—	—	T	T	T	T	T
**rs7046653**	27480967	A	A	A	A	A	A	—	—	—	A	A	A	A
rs2440622	27485418	A	—	A	A	A	—	—	—	A	A	A	A	A
**rs1977661**	27492986	C	C	C	C	C	C	—	—	C	C	C	C	C
**rs903603**	27519316	C	C	C	C	C	C	—	—	C	C	C	C	C
**rs10812610**	27523984	C	C	C	C	C	C	—	—	C	C	C	C	C
**rs2814707**	27526397	A	A	A	A	A	A	A	A	A	A	—	A	A
**rs3849942**	27533281	A	A	A	A	A	A	A	A	A	A	—	A	A
rs12349820	27543876	T	T	—	T	—	—	—	—	—	T	T	T	T
**rs10122902**	27546780	G	G	G	G	G	G	—	—	G	G	G	G	G
**rs10757665**	27547919	T	T	T	T	T	T	—	—	T	T	—	T	T
**rs1565948**	27549733	G	G	G	G	G	G	—	—	G	G	—	G	G
**rs774359**	27551049	C	C	C	C	C	C	—	C	—	C	—	C	C
**rs2282241**	27562255	G	G	G	G	G	G	—	—	G	G	G	G	G
**rs1948522**	27565785	C	C	C	C	C	C	—	—	C	C	C	C	C
**rs1982915**	27569560	G	G	G	G	G	G	—	—	—	G	G	G	G
rs7868845	27574530				T/C									
**rs2453556**	27576162	G	G	G	G	G	G	—	—	—	G	G	G	G
**rs702231**	27578731	A	A	A	A	A	A	—	—	—	A	A	A	A
**rs696826**	27579657	G	G	G	G	G	G	—	—	—	G	G	G	G
**rs2477518**	27589746	T	T	T	T/C	T		—	—	—	T	C	T	T

Haplotype deduced directly from array genotyping (this study) or haplotype given or deduced from previous publications, or haplotype derived from linkage analysis of families we have analyzed. Imputed SNP genotypes are not given. [—] indicates genotype not assessed or not clear because of ambiguous phase. The family US number 3 has not been published but has a phenotype consistent with other families with this phenotype and a lod score of > 1.2 with chromosome 9 markers. Note the discrepant results for Pearson et al. rs1444533 (centromeric) and Boxer et al. rs2477518 (telomeric) which suggest definitive flanking SNPs for the locus. SNPs included in the haplotype analysis are in bold. Key: ch9, chromosome 9; SNP, single nucleotide polymorphism.

a rs1444533 was dropped from the 20 SNP haplotype analysis.
