# Supplementary material for: The chromosome 9 ALS and FTD locus is probably derived from a single founder
Source: Neurobiol Aging. 2012 Jan;33(1):209.e3–8. doi: 10.1016/j.neurobiolaging.2011.08.005 (PMC3312749; doi:10.1016/j.neurobiolaging.2011.08.005)

Supplementary Figure 1 Mok et al.

20 SNP haplotype block as assessed in CEU Hapmap data set.

(the 140kb region is marked by the 2 arrows)


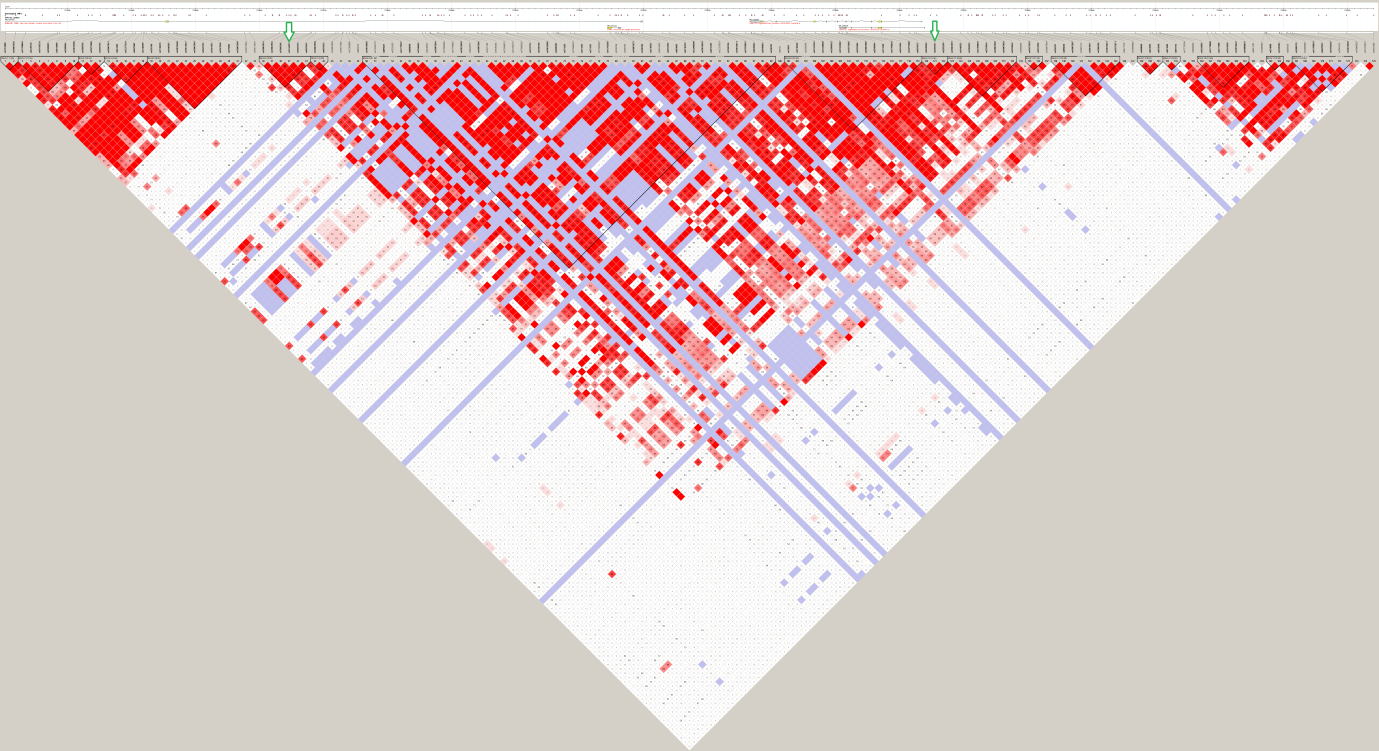

Supplement: Supplementary Figure 1 [file mmc1.doc]
